# Extracellular vesicle and particle biomarkers in cancer: a machine learning blueprint for liquid biopsy

**DOI:** 10.1186/s12951-026-04525-9

**Published:** 2026-05-30

**Authors:** Jinling Yu, Gen Xu, Yu Li, Mingyao Huang, Liang Yang, Xueqiang Peng

**Affiliations:** 1 Department of General Surgery, The Fourth Affiliated Hospital, China Medical University, Shenyang, 110032 China; 2 School of Basic Medical Sciences, Putian University, Putian, 351100 Fujian province China; 3 Shenyang Clinical Medical Research Center for Diagnosis, Treatment and Health Management of Early Digestive Cancer, Shenyang, 110032 China

**Keywords:** Liquid biopsy, Extracellular vesicles (EVs), Machine learning, Cancer diagnosis, Precision oncology

## Abstract

The combination of machine learning and liquid biopsy is rapidly promoting the development of precision cancer diagnosis. Extracellular vesicles and particles (EVPs) are liquid biopsy markers with great diagnostic value due to their unique structure, high stability and strong disease specificity in body fluids. With the rapid development of artificial intelligence (AI), machine learning enables the identification of informative biomarkers from high-dimensional, complex, and large-scale biological data. Thanks to this, EVPs have made rapid progress in the correlation research of tumor diagnosis and prognosis evaluation in recent years. This review focuses on cancer, the area in which liquid biopsy is most extensively studied and clinically needed. This review focuses on cancer as a primary application domain of liquid biopsy and provides a structured overview of machine learning methodologies in this context. We summarize recent advances in feature mining, multi-omics integration, and multi-marker fusion strategies for blood- and EVP-derived data, while also discussing key challenges, including data heterogeneity, model interpretability, and clinical validation.

**Graphical abstract:**

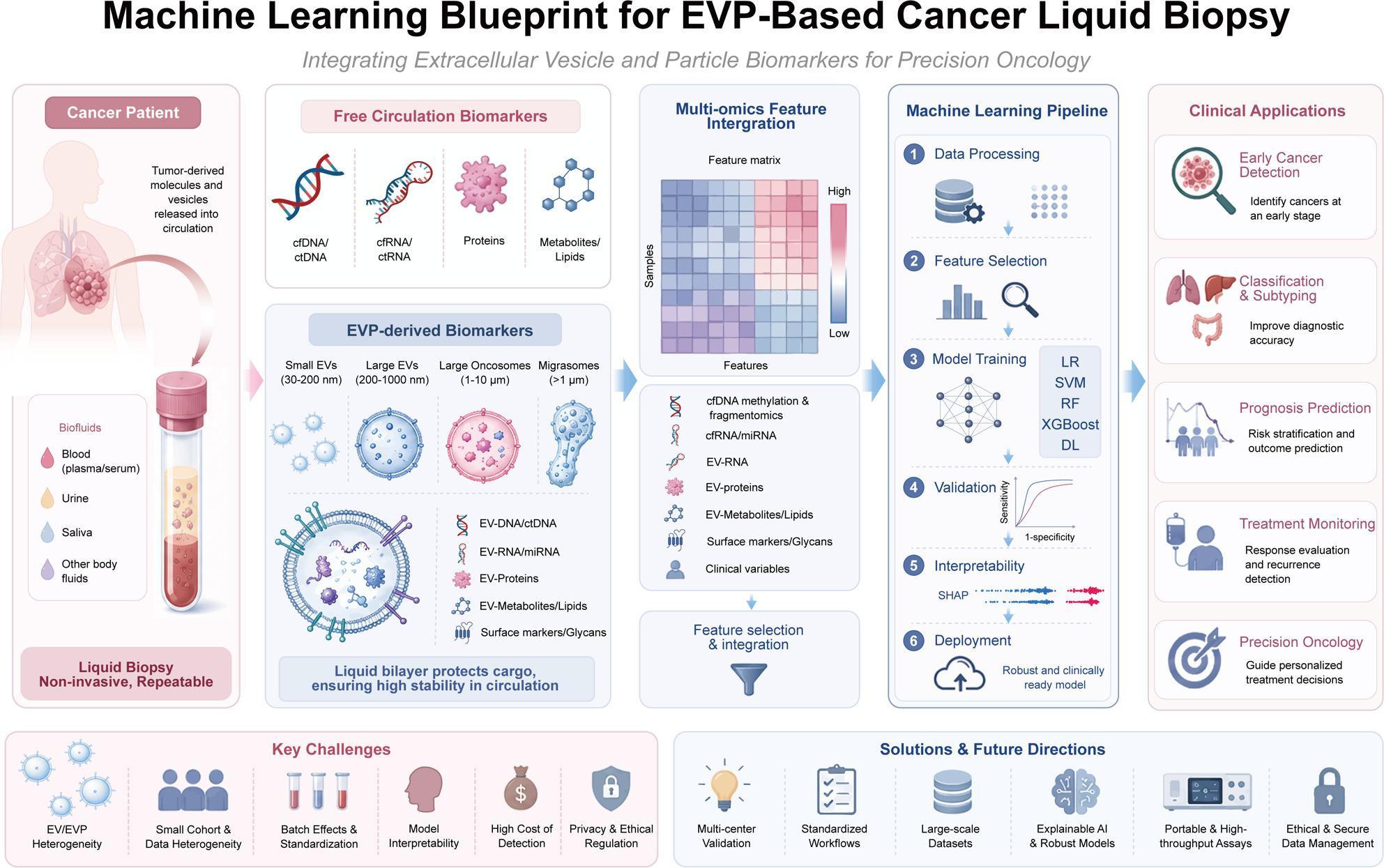

## Introduction

Diseases, most notably malignant neoplasms, function as a significant lethal factor in global public health [1]. Early cancer detection is critical for improving patient prognosis. However, the pathogenesis of malignant tumors is molecularly occult: Early tumors will have aberrant alterations at the genomic and epigenetic levels and abnormal protein expression [2, 3]. These subtle molecular changes precede morphological abnormalities, and are difficult to identify by traditional clinical diagnostic methods. These include invasive approaches represented by tissue biopsy, imaging examinations such as CT, MRI, ultrasound, and conventional diagnostic methods such as serum protein marker detection [4, 5]. The absence of early clinical signs often delays patients’ presentation to healthcare providers until overt symptoms emerge. By this time, the disease has frequently advanced to intermediate or late stages (clinical stage Ⅲ-Ⅳ), and the optimal window for clinical intervention has been missed. This represents one of the core causes of poor prognosis in patients with cancer [6–9]. Although conventional diagnostic methods yield high accuracy, they have critical limitations. Tissue biopsy is invasive and difficult to achieve dynamic monitoring of disease through repeated sampling [10, 11]. Most imaging examinations require repeated testing and may impose substantial economic burdens on patients [12]. Furthermore, their diagnostic performance relies on physicians’ expertise, especially for early lesions or atypical lesions, the detection rate is low, often showing insufficient diagnostic accuracy [13, 14]. These bottlenecks have motivated researchers and clinicians to pursue more convenient, patient-centered alternatives.

**Fig. 1:**
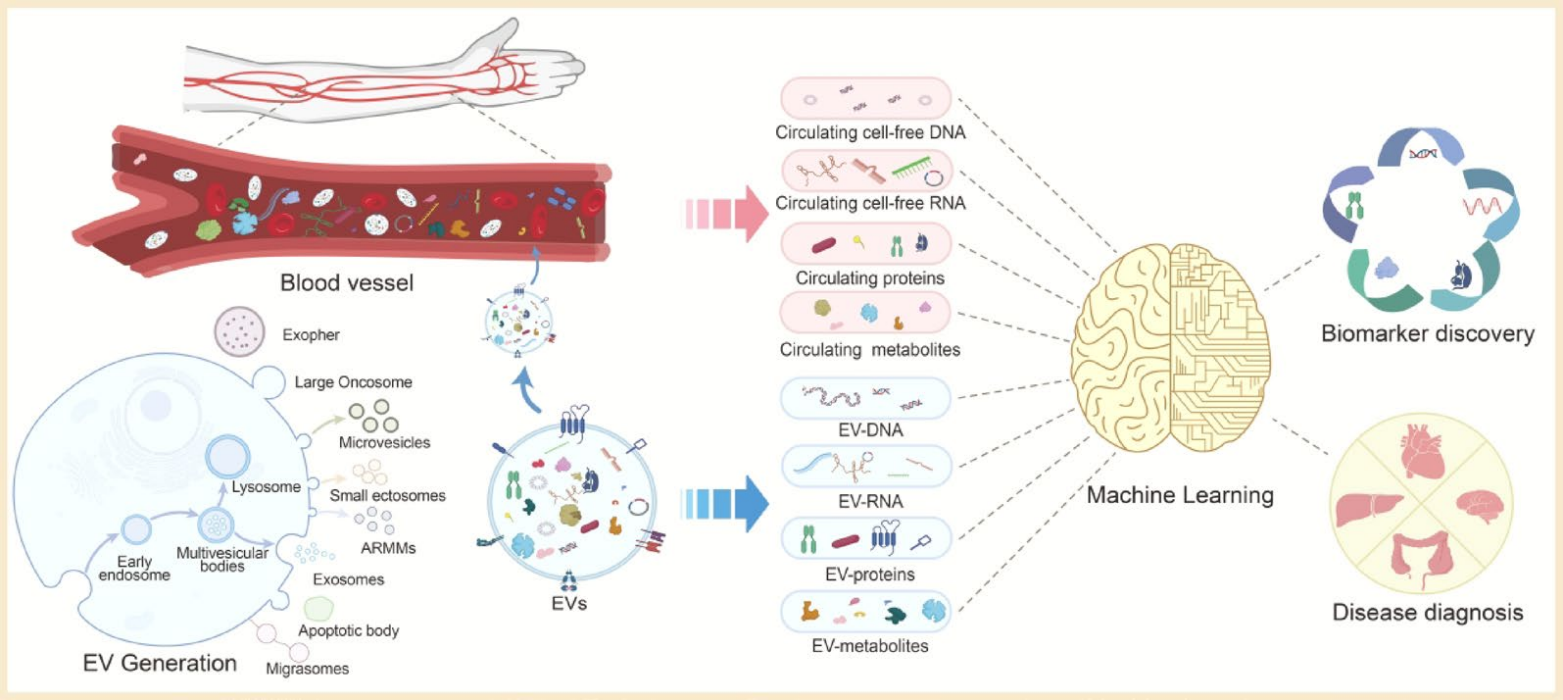
This figure shows workflow of liquid biopsy and machine learning-based disease diagnosis. EV biogenesis and circulating biomarkers (cell-free and EV-derived multi-omics data) feed into machine learning models for biomarker discovery and multi-organ disease diagnosis

The above-mentioned bottlenecks have driven the rapid development of liquid biopsy as a noninvasive alternative. By analyzing specific molecules (DNA, RNA, protein, metabolites, etc.) released into blood and other body fluids during the disease process, liquid biopsy has the advantages of rapid, convenient, and repeatable dynamic monitoring, and has shown great potential in early tumor screening and course management [15–17] (Fig. 1).

However, commonly used clinical liquid biopsy biomarkers also have limitations. Prostate-specific antigen (PSA) is sensitive for prostate cancer screening but leads to frequent false positives in benign prostatic hyperplasia and misses cases with low PSA expression [18–21]. Alpha-fetoprotein (AFP) exhibits high specificity for advanced hepatocellular carcinoma yet has low sensitivity for early-stage disease and false positives in benign liver disorders [9]. Carcinoembryonic antigen (CEA) is applicable for follow-up of gastrointestinal cancers [22–24] but lacks specificity as it is elevated in other tumors [25] and inflammatory diseases [26].

The above challenges point the way to future research directions. On the one hand, searching for more excellent novel biomarkers based on high-throughput histology technology [27, 28]; on the other hand, constructing a diagnostic decision model through multi-marker combined detection to achieve more efficient disease screening and accurate diagnosis [29, 30]. The realization of both strategies relies on deep mining and parsing of massive and complex biomedical data.

Compared between traditional statistical analysis methods and machine learning (ML) algorithms, there is no absolute advantage or disadvantage. When dealing with high-dimensional, nonlinear and multimodal data, the former suffers from insufficient feature mining and limited model fitting capability, while the latter has its advantages through feature engineering and nonlinear mapping. But in the data with small sample size, low dimension and clear prior statistical rules (such as single serum marker detection [31], traditional statistical methods may have better stability and interpretability [32–35].

## The evolution of machine learning

### Traditional machine learning algorithm

**Fig. 2:**
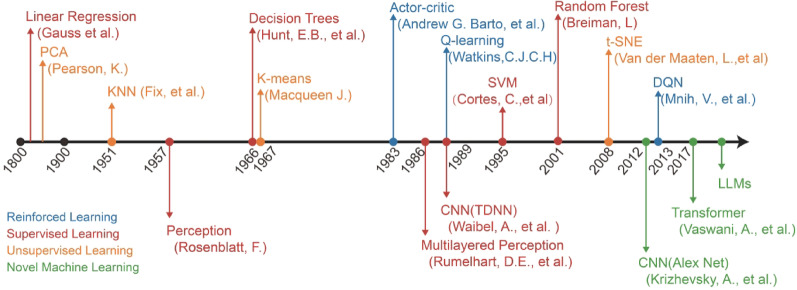
This figure illustrates the chronological evolution of major machine learning methods from the 19th century to the present. The horizontal axis represents time, while the vertical listings show representative models and their proponents, including Linear Regression, Perceptron, Multilayer Perceptron, Support Vector Machines, Random Forest, Convolutional Neural Networks (e.g., AlexNet), Transformers, and Large Language Models (LLMs)

**Fig. 3:**
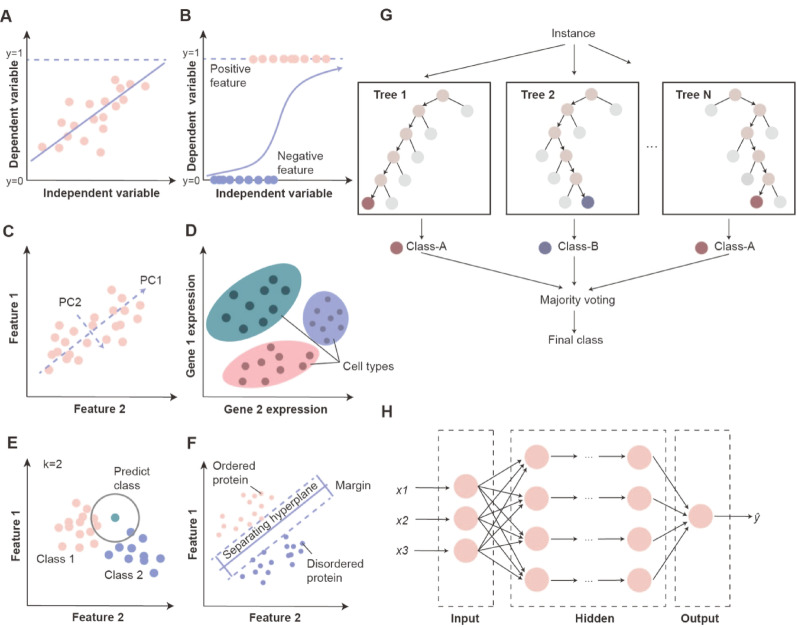
This figure illustrates the basic principles of various machine learning models and their applications: **A**. Logistic regression models the relationship between predictor variables and binary class labels; **B**. Ensemble decision trees (e.g., random forests) predict sample classes via majority voting across multiple decision trees; **C**. Principal component analysis (PCA) reduces the dimensionality of gene expression data to distinguish cell types; **D**. Support vector machines (SVMs) classify ordered and disordered proteins by constructing a maximum-margin separating hyperplane; **E**. K-nearest neighbors (k-NN) algorithm classifies samples based on proximity in feature space (here, k=2); **F**. Neural networks learn complex nonlinear mappings between input variables and output predictions via multiple hidden layers.

The development of ML can be traced back to the mid-20th century. In 1950, Alan Turing proposed the Turing test in Computing Machinery and Intelligence [36], which laid the theoretical foundation for the birth of ML. Then it went through many stages of development (Fig. 2) such as symbolism, connectionism and deep learning, and finally formed three core paradigms of supervised learning, unsupervised learning and semi-supervised learning [37–41] (Fig. 3).

Supervised ML involves fitting a model to labelled data (or its subsets), where there exists some ground-truth lables, which is usually experimentally measured or assigned by humans [42]. In contrast, unsupervised learning does not need to manually label the data, but based on the unlabeled data, it realizes clustering, dimensionality reduction and other tasks by mining the internal structure and potential rules of the data [43, 44].

Semi-supervised learning is an important paradigm between supervised learning and unsupervised learning, which combines a small amount of labeled data with a large amount of unlabeled data for model training. It not only solves the high cost of labeled data for supervised learning, but also improves the performance of unsupervised learning models. In the early diagnosis of cancer, semi-supervised learning can use a large number of healthy unlabeled samples to optimize the model to improve the sensitivity [45, 46].

Reinforcement learning (RL), as a unique branch of ML, has been applied in the field of cancer research and oncology. However, owing to the absence of explicit reward-feedback mechanisms in liquid biopsy datasets, it is difficult to obtain the “decision-reward” data required for its training, and it is still mainly used in exploratory applications [47, 48]. 

### Novelmachine l earning algorithms

 Deep learning, a multi-layer neural network-based subset of ML, is a powerful tool for complex biomedical data modeling [45, 49]. Unlike traditional ML relying on manual feature engineering, its core strength is end-to-end automatic feature extraction without predefined rules.

Deep learning models are more complex and have significant advantages in scenarios with large samples and high-quality datasets. For this reason, in scenarios with limited samples, high model complexity can easily lead to overfitting [50, 51]. Therefore, algorithm selection must be data-adaptive. For small-sample liquid biopsy studies, traditional algorithms or model fusion strategies are often preferable. The recommended practical strategy includes training multiple algorithms and selecting the best performer based on rigorous multi-cohort validation, rather than defaulting to the most complex model [45, 52, 53]. It has been demonstrated that deep learning models may even perform worse than simple linear regression in biological data settings more complex than liquid biopsy [54, 55]. Each ML paradigm includes diverse model variants, and as we mentioned before—— no single algorithm consistently outperforms others across all liquid biopsy datasets. An empirical, trial-and-error strategy is therefore more prudent. The optimal approach is to train and optimize multiple candidate methods, select the best performer on the validation set, and conduct a final comparison on an independent test set [45].

### Performance metrics for liquid biopsy ML models

To understand the performance of these models, key evaluation metrics for classification models are detailed in Tables 1 (1). The performance of “one pass and one failure” reflects the distribution of the ability of the model in different decision-making dimensions, and defines its clinical applicability boundary.

**Table 1:** Some key evaluation metrics for machine learning models

Name	Concept	Formula				Core Significance	Clinical Application in Liquid Biopsy
Confusion Matrix	Tabular summary of model classification results			Actual Values		The only non-numerical index, the “original result carrier” rather than the “efficiency quantitative value”, the premise of many quantitative index calculation and model performance analysis, directly reflecting missed diagnosis/misdiagnosis risks	No direct clinical application; serves as the foundational data source for calculating all quantitative metrics, helping researchers visualize missed diagnosis (FN) and misdiagnosis (FP) risks to optimize model parameters
				Negative	Positive		
		Predicted Values	Negative	TN	FP		
			Positive	FN	TP		
Accuracy	Proportion of correct predictions among total predictions	(TP + TN)/(TP + TN+FP + FN)				Reflects the overall prediction accuracy of the model and is suitable for balanced data sets	Rarely used as a primary metric in cancer liquid biopsy; more applicable for small-scale, balanced cohorts
Precision	Proportion of correctly predicted positives among all predicted positives	TP/(TP + FP)				Reflects the proportion of true positives among samples predicted to be positive and is applicable to reduce the false positive rate	Prioritized in population-wide cancer screening or post-screening confirmation to minimize false positives
Recall/ Sensitivity	Proportion of correctly predicted positives among all actual positives	TP/(TP + FN)				Reflects the proportion of true positive samples that are correctly predicted and is suitable for reducing the false negative rate	Critical for early cancer detection where minimizing false negatives is paramount
Specificity	Proportion of correctly predicted negatives among all actual negatives	TN/(TN + FP)				Reflects the proportion of true negative samples that are correctly predicted and is complementary to recall	Complementary to Recall in clinical decision-making; used to rule out cancer in patients with ambiguous symptoms and reduce overdiagnosis
F1 Score	Harmonic mean of precision and recall	2 × (Precision × Recall)/ (Precision + Recall)				The harmonic mean of precision and recall is suitable for comprehensive evaluation of imbalanced data sets	Supplementary metric for scenarios requiring balanced false positive/false negative risks
AUROC	A performance metric for binary classification models	∫₀¹ TPR(FPR) d(FPR)				It comprehensively reflects the diagnostic efficacy of the model under different thresholds, which is the core index of liquid biopsy model evaluation, and is not affected by sample balance	Gold-standard primary metric for model validation and clinical translation; robust to class imbalance and threshold selection, enabling objective comparison of different models across multi-center cohorts; core for regulatory approval
C-Index	A metric evaluating the concordance of survival prediction models in ranking patient prognosis	Number of Concordant Pairs/ Number of Usable Pairs​				Gold-standard for tumor prognostic stratification; ranges from 0.5 (random) to 1.0 (perfect prediction), quantifying the model’s ability to rank patient survival risk	Exclusive for prognostic stratification and treatment decision-making in advanced cancer patients
TP (True Positive): Correctly predicted positive samples. FP (False Positive): Negative samples incorrectly predicted as positive. TN (True Negative): Correctly predicted negative samples. FN (False Negative): Positive samples incorrectly predicted as negative. AUROC: Area Under the Receiver Operating Characteristic curve.							

AUROC passing but Accuracy failure is a typical and acceptable metric imbalance in cancer liquid biopsy. This indicates that the model can effectively distinguish cancer from healthy samples. However, due to the high proportion of healthy samples, even if the model correctly identifies all cancer samples, its overall accuracy will still be low due to the large number of healthy samples. In this case, the low accuracy is not a defect of the model, but an inevitable result of the data distribution fully adapting to the characteristics of the cancer screening scene. It has been reported that in cancer early screening, high AUROC values are the core of the value of the model, and Accuracy is highly misleading in such imbalanced data sets [56]. However, F1 Score passing but AUROC failure can reveal the insufficient generalization ability of the model, indicating that the model only performs well under a specific threshold, but the ranking ability in the full threshold range is weak, and there is likely to be overfitting. Only relying on the F1 score under a single threshold will mask the instability of the model. As a threshold-independent index, AUROC can better reflect the true discrimination ability of the model [57].

**Fig. 4:**
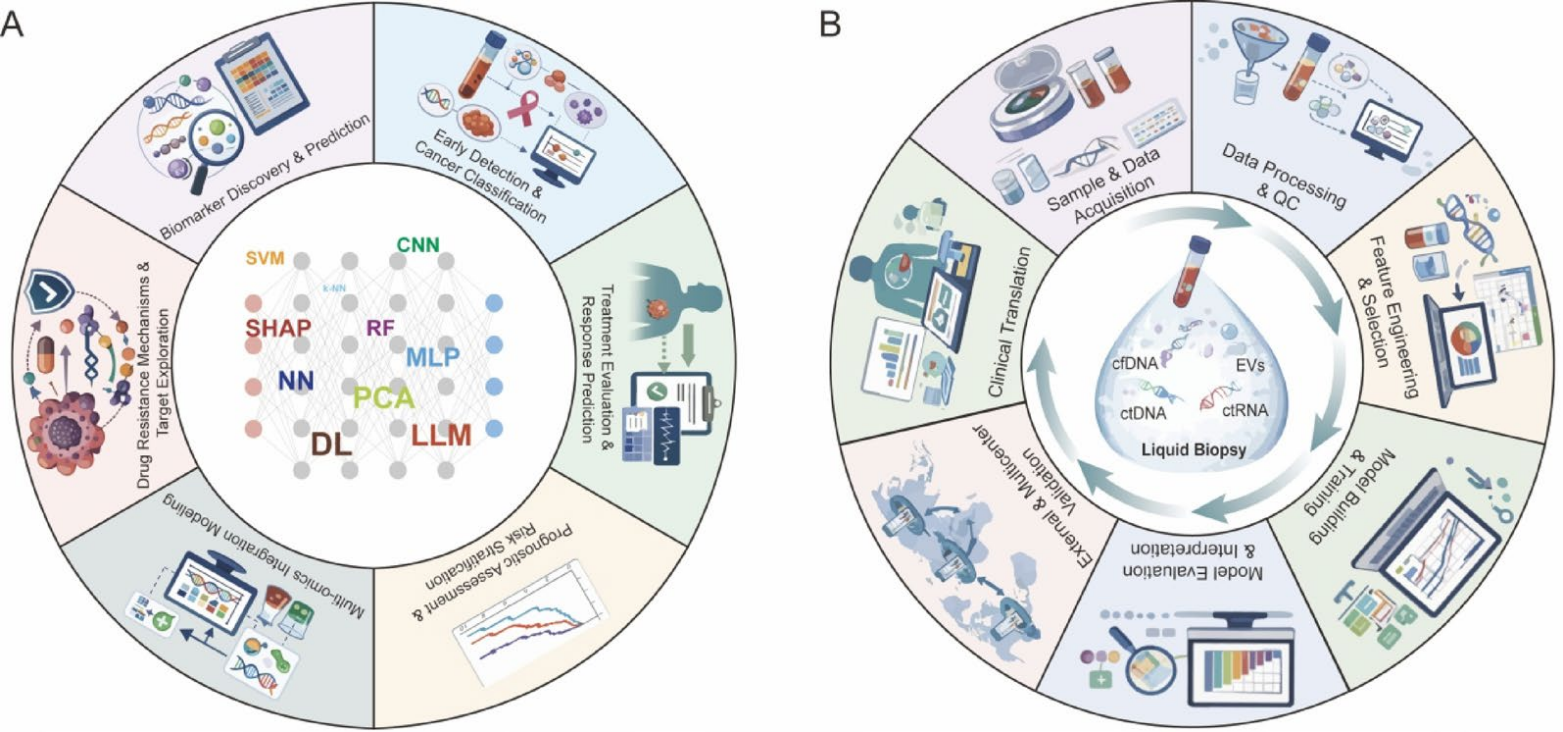
This figure provides a schematic overview of the analytical role of artificial intelligence (AI) and machine learning (ML) in liquid biopsy–based cancer research. **A**. The representative AI/ML algorithms (e.g., SVM, CNN, RF, DL, LLM, SHAP) and their six key application domains in liquid biopsy; **B**. The end-to-end translational workflow for AI/ML-driven liquid biopsy diagnostics, covering sample acquisition, data processing, feature selection, model development, validation, and clinical translation.

To provide a clear overview of ML algorithms and their core applications in cancer liquid biopsy, we summarize representative models and key clinical application cases in Fig. 4. This figure provides a roadmap for subsequent application discussions.

## Machine learning applications for circulating free markers

Blood serves as a key circulating medium that carries dynamic molecular signatures reflecting systemic physiological and pathological status. This section reviews ML applications in cancer liquid biopsy, focusing on circulating free biomarkers and extracellular vesicles (EVs). Representative studies in this field are summarized in Tables 2 (2). It should be noted that circulating tumor cells (CTCs) and tumor-educated platelets (TEPs) themselves or their bioactive cargoes also present considerable diagnostic value and potential [58–60], but they are not discussed in this review.

**Table 2:** Some representative machine learning-based diagnostic biomarker studies in cancer

Omics	Model Function	Biomarkers	Sample Size (Training: Validation)	Machine Learning Algorithms	Model Function (Validation Cohort Focused)	Ref.
RNA Omics	Early diagnosis, cancer staging of PDAC	FGA, KRT19, HIST1H2BK, ITIH2, MARCH2, CLDN1, MAL2, TIMP1	188:313	LASSO, RF, SVM	Cancer Diagnosis: AUC = 0.950 (Internal), AUC = 0.936 (External) Cancer Staging: Internal: AUC = 0.978 (Stage I), 0.980 (Stage II), 0.927 (Stage III/IV) External: AUC = 0.895 (Stage I), 0.929 (Stage II), 0.972 (Stage III/IV) AUC = 0.949 (Stage I/II Combined)	[246]
	Early diagnosis of gastric cance	RP11.443C10.1, CTD-2339L15.3, LINC00567, DGCR9	978:227	LASSO-LR, LR, XGBoost, RF, KNN, SVM, Naive Bayes	AUC = 0.942 AUC = 0.949 (External)	[231]
	Early diagnosis of colon cancer	Let-7b-3p, miR-145-3p, miR-139-3p	25:134	LR	AUC = 0.927	[195]
	Early diagnosis of CRC, identification of precancerous lesions	CRC diagnosis model (8):Let-7f-5p, C19orf43, TOP1, PPDPF, lnc-MKRN2-42:1, LNC-EV-9572, HIST2H2AA4, miR-320a-3p AA diagnosis model (9): miR-425–5 p, Let-7f-5p, C19orf43, TOP1, PPDPF, LNC-EV-9572, lnc-MKRN2-42:1, HIST2H2AA4, MT-ND2	60:123	Lasso regression, LR, SVM	AUC = 0.8 (CRC) AUC = 0.88 (AA)	[188]
	Diagnosis of liver cancer	HNRNPA3P5, PTMAP2, HMGN1P38, chr17:4792554147927724:+(SP2), chr12:30122323017398:+(TEAD4), chr17:500391615003998:+(KIF1C), chr7:100805321100805501:-(EPHB4), chr19:4494853344948701:+(APOC2)	122:80	RF, LASSO, SVM	AUC = 0.9825	[247]
Proteomics	Diagnosis of colorectal cancer	PF4, AACT, CEA, CA19-9	338:574	RF, Rpart, LR, KNN, SVM	AUC = 0.883 AUC = 0.810 (External)	[248]
	Early diagnosis of CRC, identification of precancerous lesions	FIBG, PDGF-β, TGF-β CEA	239:165	NNET, LDA, Glmnet, LR, Naive Bayes, CV-Glmnet, KNN, QDA, SVM, RF, XGBoost, Rpart	AUC = 0.937	[53]
	Diagnosis of colorectal cancer	APOA4, SEROINA3, GPX3, SNCA, YKT6, RAN, ENPP2, ANTXR1, THBS4, SPP2	21:9	Classification tree, KNN, RF, Naive Bayes, SVM, Backpropagation Neural Network	AUC = 1.00 (Polyp vs. HC), 0.97 (CRC vs. HC), 0.94 (CRC vs. Polyp)	[50]
	Diagnosis of hepatocellular carcinoma	KNG1, F11, KLKB1, CAPNS1, CDH1, CPN2, NME2	96:96	RF, LR, Linear SVM, RBF SVM, Decision tree, AdaBoost	AUC = 0.978 (HCC vs. CHB), 0.943 (HCC vs. LC), 0.953 (HCC vs. nonHCC)	[241]
	Early diagnosis of hepatocellular carcinoma	EV subpopulations (3): EpCAM+ CD63 + EVs, CD147 + CD63+ EVs, GPC3 + CD63+ EVs	106:72	LR	AUC = 0.93	[249]
Metabonomics	Diagnosis of hepatocellular carcinoma	m/z features(6): 138.89, 138.08, 140.06, 142.91, 140.90, 462.00	146:146	LR, RF, SVM, NN, kNN, Decision tree, OPLS-DA	AUC = 1 (HC vs. LC), 1 (HC vs. HCC), 1 (HCC vs. LC)	[211]
Multi-omics	Early diagnosis, cancer staging of PDAC	cf-miRNAs(5): miR30c-5p, miR340-5p, miR335-5p, miR23b-3p, miR142-3p and candidate exo-miRNAs(8): miR145-5p, miR200b-3p, miR429, miR1260b, miR145-3p, miR216b-5p, miR200a-3p, miR217-5p	96:95	LR	Cancer Diagnosis: AUC = 0.93 Cancer Staging: AUC = 0.93 (Stage I/II), 0.94 (Stage III/IV)	[214]
	Early diagnosis of PDAC, metastasis prediction	Early diagnosis model (5): EV-CK18 mRNA, EV-CD63 mRNA, EV-miR-409, ccfDNA concentration, CA19-9 Metastasis prediction model (4): EV-miR-1299, EV-GAPDH, Circulating mutant KRAS allele fraction, CA19-9	Diagnosis: 47:136 Metastasis: 20:37	LASSO, KNN, SVM, Linear discriminant analysis, LR, Naive Bayes	Cancer Diagnosis: AUC = 0.95	[213]
	Benign/malignant lung tumor differentiation	10 clinical features, 3 cancer protein markers, cfDNA mutations (8 features), cfDNA methylation (30 MCBs)	98:29	SVM, BNB	AUC = 0.86	[217]
	Early diagnosis of ESCC	Dual-feature model (2): MMP13 and SCC Five-feature model (5): SCC, Age, MMP13, CEA, NSE	414:1384	LR, LASSO, Ridge regression, RF, SVM, NN	External Dual-Feature Model: HNCH: AUC = 0.93 (ESCC), 0.92 (Early ESCC) CHSUMC: AUC = 0.98 (ESCC), 0.98 (Early ESCC) Prospective Dual-Feature Model: SYSUCC II: AUC = 0.88 (ESCC), 0.78 (Early ESCC) HNCH II: AUC = 0.86 (ESCC), 0.84 (Early ESCC) CHSUMC II: AUC = 0.97 (ESCC), 0.96 (Early ESCC) Prospective Five-Feature Model: AUC = 0.90 (SYSUCC II), 0.89 (HNCH II), 0.98 (CHSUMC II)	[137]

In liquid biopsy, the classification model dominates. Its performance is most comprehensively evaluated with the area under the ROC curve (AUROC) as the preferred metric due to its robustness to class imbalance and its powerful ability to rank samples with all classification thresholds [61]. AUROC (Area Under the ROC Curve) will be abbreviated as AUC when it appears below. All performance measures reported here are from the validation cohort, unless otherwise stated. 

### Machine l earning applications based on circulating cell-free DNA (cfDNA)

 Circulating cfDNA is passively released into the peripheral circulation through cellular apoptosis and necrosis [62]. Its tumor-derived fraction, circulating tumor DNA (ctDNA) [63], though scarce, carries cancer-specific alterations that are vital for early detection, identification of minimal residual disease after surgery, and recurrence risk assessment when screening high-risk populations [64–70].

For low-dimensional cfDNA features such as single mutation panels, small-scale methylation signatures, regularized linear models remain optimal [71–73]. In small-sample, low-noise settings, simple linear approaches have been reported to outperform more complex deep learning models [55]. In contrast, for high-dimensional data types—including fragmentomics, whole-genome methylation, and multimodal cfDNA profiles—tree-based methods (RF, XGBoost) and shallow neural networks are often preferred, as they can capture non-linear feature interactions while maintaining robustness in limited clinical datasets [74–76].

ML plays a crucial role in extracting informative signals from low-abundance ctDNA. ctDNA-based ML models have demonstrated improved performance over traditional approaches in cancer diagnosis and complication risk prediction [77–79]. However, the limited proportion of ctDNA present in early-stage cancers may affect sequencing stability [80]. To address this, recent studies increasingly integrate advanced ML strategies with improved sequencing technologies to enhance detection reliability [65]. Deep learning models, particularly when applied to large-scale or multimodal datasets, show potential to better capture complex molecular patterns, thereby improving early cancer detection and classification [81, 82]. A major challenge remains the discrimination of true tumor-derived mutations from alterations caused by clonal hematopoiesis, a key source of false positives [62, 83].

Given the limitations of mutation-based approaches, epigenetic markers such as DNA methylation (5-methylcytosine and 5-hydroxymethylcytosine) provide valuable alternatives due to their early and frequent alterations during carcinogenesis [84–90]. Methylation-based models, including those leveraging differentially methylated regions (DMRs) and differentially hemimethylated regions, have achieved high diagnostic accuracy (AUC > 0.94) across multiple cancer types, often outperforming conventional biomarkers such as CEA [91–93].

cfDNA fragmentomics—analyzing features like Fragment Size Ratio (FSR), Fragment Size Distribution (FSD), Mutation Context (MC), End Motif (EDM), and BreakPoint Motif (BPM)—provides a rich source of diagnostic information [94–97]. Stacking models that integrate multiple fragmentomic features and ML algorithms have shown exceptional performance in early cancer detection, outperforming standard protein biomarkers across multiple cancer types [98–104]. This approach is also being validated at a pan-cancer level [97, 105] and extended to mitochondrial cfDNA (cf-mtDNA) for applications like hepatocellular carcinoma diagnosis. cfDNA fragmentomics is paving the way for highly accurate, multimodal pan-cancer screening [95, 106, 107]. A critical technical consideration is that model performance depends on sequencing depth, so it is advisable to evaluate performance at various depths [79, 81, 99, 102, 103].

### Machine learning applications based on circulating cell-free RNA

Circulating cfRNA provides a dynamic snapshot of gene regulation, complementing the static genomic information from cfDNA. The cfRNA pool is diverse, including microRNAs (miRNAs), long non-coding RNAs (lncRNAs), circular RNAs (circRNAs), and other novel RNA species [30].

Among these, miRNAs are the most extensively studied due to their high stability in circulation and their role as key post-transcriptional regulators [108–112]. ML models using miRNA expression profiles have shown strong diagnostic performance across various cancers, including esophageal adenocarcinoma and prostate cancer, as well as large-scale pan-cancer studies [113–116].

In particular, the sample type affects circulating miRNA content [117]. Due to non-specific miRNA release during coagulation, plasma may serve as a more reliable carrier for tumor-derived circulating miRNAs [112, 118].

Beyond miRNAs, other non-coding RNAs (ncRNAs) have shown diagnostic potential for cancers like colorectal cancer and hepatocellular carcinoma [119–122], while novel RNA classes such as tRNA-derived small RNAs (tsRNAs) and orphan non-coding RNAs (oncRNAs) are increasingly integrated into models, with some outperforming traditional miRNA-based approaches in specific contexts [123–127].

The advancement of cfRNA-based diagnostics is closely linked to technological innovations. New sequencing methods (e.g., SLiPiR-seq) enable the analysis of RNA modifications and reveal that combining multiple cfRNA types enhances diagnostic accuracy beyond any single class [128]. Concurrently, novel extraction-free assay platforms are being developed to streamline workflow and improve reliability, facilitating clinical translation [129].

A major challenge is the instability of long RNAs like mRNAs and lncRNAs in circulation, which may lead to data quality distortion, reduced feature reliability, and impaired model generalization ability [130].

### Machine learning applications based on circulating proteomics

Proteins are key players in life activities that reflect the physiopathological state of an individual, and therefore most of the hematological biomarkers commonly used in clinical practice today are proteins [131]. Advances in mass spectrometry (MS) have dramatically expanded the breadth of the detectable proteome, generating high-dimensional and complex datasets that are challenging to interpret via traditional analytical methods [132]. ML mines integrated protein signatures from high-dimensional data, enabling a shift from single-marker detection to multi-feature modeling [133–136]. The models constructed by MS-ML collaboration are superior to single biomarkers in early cancer detection and risk stratification [137, 138].

An emerging strategy is tissue-informed proteomics, where protein profiles from diseased tissues guide plasma biomarker discovery. By integrating tissue and circulating data, ML models can capture biologically relevant signals related to tumor subtype and therapeutic response. This cross-domain integration improves both predictive performance and biological interpretability [139, 140].

Another emerging strategy is mutual validation of multiple liquid-biopsy markers to improve diagnostic performance and compensate for the shortcomings of single-source samples. For example, by verifying the consistent mRNA markers of urine and plasma EVs and integrating the data to construct a linear discriminant analysis (LDA) model, the healthy individuals and bladder cancer patients were completely distinguished [141].

Challenges such as batch effects, wide dynamic range of plasma proteins, and technical variability remain, which may affect model robustness and reproducibility [142, 143].

### Machine learning applications based on circulating metabolomics

Circulating metabolomics captures the end products of cellular processes, providing a functional readout of physiological and pathological states through “circulating metabolic fingerprints” (CMFs). Increasing evidence supports the diagnostic and prognostic utility of plasma metabolites, with specific metabolite panels demonstrating strong performance in cancers such as hepatocellular carcinoma and early-stage gastric cancer, in some cases exceeding that of traditional protein biomarkers [144–146].

Lipidomics, as a major component of metabolomics, has attracted particular attention due to the role of lipid metabolic reprogramming in tumorigenesis [147, 148]. The reprogramming of lipid metabolism in tumorigenesis has prompted its investigation as a diagnostic biomarker, and its multi-omics model maintains high accuracy in patients even when one of the indicators is negative [149, 150]. More importantly, the value of lipidomics has gone beyond simple diagnosis: it can define metabolic subtypes of gastric cancer with different prognosis through cluster analysis [151], highlighting the multidimensional value of lipidomics in cancer diagnosis and treatment.

### Adaptability logic of machine learning algorithms to free blood marker data

Model selection should be guided by data dimensionality, sample size, and signal complexity.

For cfDNA data, which is typically high-dimensional with low signal-to-noise ratios, algorithms capable of capturing complex feature interactions—such as ensemble methods and, in large-scale settings, deep learning—can be advantageous. However, in small-sample scenarios, simpler models often remain more robust due to reduced overfitting risk.

Circulating proteomic data is often characterized by moderate dimensionality but limited sample sizes due to high experimental costs. In this context, traditional ML approaches with strong feature selection and regularization capabilities are generally more suitable, offering improved interpretability and robustness.

cfRNA data present additional challenges due to their instability and heterogeneity across RNA subtypes. Hybrid strategies that combine feature selection with integrative modeling are therefore commonly employed to balance noise reduction and information retention.

In contrast, circulating metabolomic data are typically lower in dimensionality but exhibit dynamic concentration changes. As a result, conventional ML algorithms are often sufficient to achieve effective modeling while maintaining computational efficiency.

## Hematologic markers in EVs

EVs are lipid bilaye-enclosed particles released by cells that facilitate intercellular communication by transferring bioactive molecules—including DNA, RNA, proteins, and lipids—from donor to recipient cells, thereby influencing processes such as immune regulation and tumor progression [152–157]. Their molecular cargo reflects the physiological state of the parent cell, making EVs promising liquid biopsy targets for cancer diagnosis and monitoring [158–161]. EVs exhibit notable stability in circulation, as their lipid membranes protect contents from degradation [162], and they are abundantly present in diverse biofluids such as blood, urine, and saliva [163, 164].

### The heterogeneity landscape of EVs and Its diagnostic implications

The potential of EVs in liquid biopsy is closely tied to their significant heterogeneity, which presents both challenges and opportunities [165]. The biological heterogeneity of EVs—specifically vesicle size, molecular composition, and pathological enrichment of different EV subtypes—provides a robust, multi-dimensional foundation for ML-driven biomarker discovery.

EVs are commonly classified based on size into small EVs (sEVs, < 200 nm) and large EVs (lEVs, > 200 nm), along with emerging types like large oncosomes (LOs) and migrasomes, each with unique origins and cargo profiles [161, 166].

The heterogeneity of EVs poses significant technical challenges. Traditional separation methods such as ultracentrifugation and size exclusion chromatography (SEC) are difficult to achieve high-purity separation [167, 168], and different subtypes have different responses to hypoxia, inflammation and other stimuli [169–171]. Emerging microfluidic, single-particle imaging, and immunoaffinity capture technologies provide new approaches for high-resolution EV analysis [172–174]. At the same time, different methods for characterizing exosomes will also directly affect the composition of exosomes and the detection of molecular load, which will further affect the construction and results of exosome-based ML models [175].

**Fig. 5:**
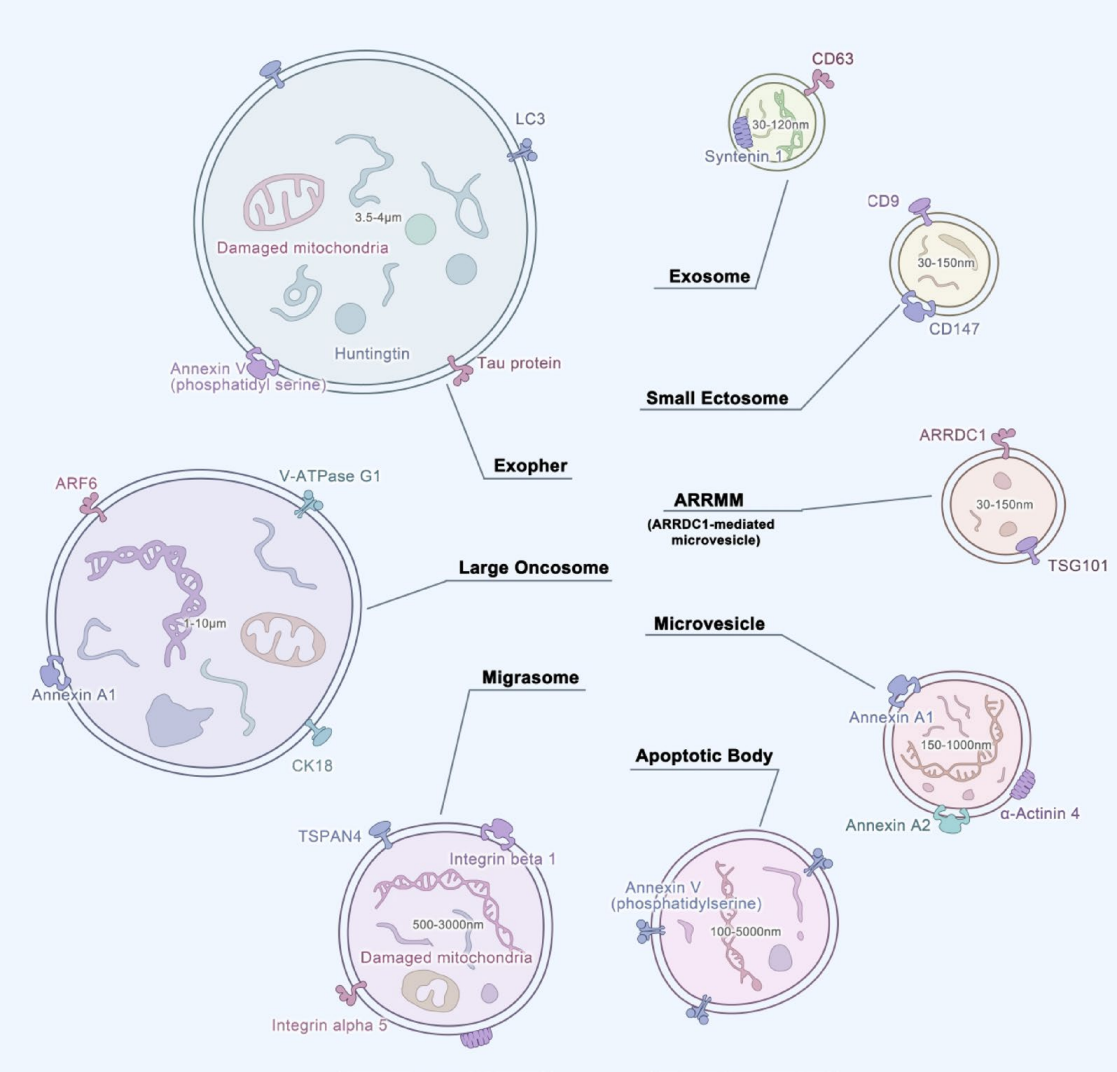
This illustration depicts the size ranges, surface markers, and cargo characteristics of different EV subpopulations and related structures. These vesicles vary widely in biogenesis, molecular content, and biological function, reflecting the complex heterogeneity of extracellular vesicle subtypes

Each EV subtype shows a specific distribution under pathological conditions (Fig. 5): sEVs in pancreatic cancer are enriched in early diagnostic markers such as Glypican-1 [176, 177]; while lEVs/LOs are more likely to carry tissue factor and mutated DNA [178, 179]. EpCAM+CD147 + tumor-associated microparticles (taMPs) can detect a variety of cancers [180]. Specific cargoes and surface markers help to target and isolate EVs, similar to feature selection in ML, where irrelevant or interfering variables are eliminated before model training, resulting in more robust and generalizable results.

### Machine learning applications based on circulating EV-DNA features

DNA, one of the major cargoes of EVs, is diverse and includes both genomic DNA (gDNA) and mitochondrial DNA (mtDNA) [181]. The role of EV-DNA as a potential biomarker for cancer has been reported in the literature [182, 183]. EV-DNA has the same potential as a biomarker due to its bilayer lipid membrane encapsulation, which is not easily degraded [184–187]. However, at present, relevant research based on EV-DNA combined with ML algorithms is still limited and the related field remains to be explored [183].

### Machine learning applications based on circulating EV-RNA features

EVs carry a large number of RNA molecules (including mRNA, miRNA, lncRNA, etc.) inside, and their phospholipid bilayer membranes can effectively protect the internal RNAs from degradation by RNases in the environment, making EV-RNA a relatively stable target for detection. In recent years, studies targeting EV-RNA have covered a variety of malignant tumors [27–29, 34, 188].

Plasma-derived EVs also contain long RNAs (mainly mRNAs, lncRNAs, and circRNAs) and influence tumor initiation and progression [189, 190]. Therefore, EV long RNA (exLR) differential expression profiles may be potentially valuable for distinguishing cancer patients from healthy individuals. Similarly, the value of EV mRNA in tumor diagnosis and treatment has been validated by several studies [191, 192]. Non-coding RNAs (ncRNAs) make up the vast majority of RNAs transcribed in the human genome [193], which has been the primary focus of RNA marker research for liquid biopsy. EV-derived miRNAs have been shown to have diagnostic performance exceeding that of free plasma miRNAs. However, conclusions may be limited by sample size and need to be confirmed in larger samples as well as a wider range of sample sources [194, 195]. It is noteworthy that multiple RNAs were included in the features, which to some extent indicates that multiple types of RNAs have a synergistic optimization effect in improving the predictive performance of the models.

In addition, some novel research directions are gradually emerging, such as differential editing of post-transcriptional RNA, alternative splicing (AS), and alternative polyadenylation (APA). Studies have been conducted to detect editing sites in miRNAs of circulating sEVs, which offers the potential for non-invasive differentiation between normal and tumor samples based on blood [196].

### Machine learning applications based on circulating EV-protein features

EV protein composition is more complex, and it is generally accepted that EVs contain both non-specific and specific proteins. The former, such as CD9, CD63, CD81, and other EV markers, are commonly found, while the latter is found in EVs generated from specific cellular sources or specific pathological processes [197]. Thus, the presence of non-specific proteins can help to trace EVs back to their cell of origin.

As the original intention of single-cell technology development was to search for subpopulation or even individual specificity hidden below the population mean, researchers gradually began to focus on protein analysis techniques at the level of individual EVs, such as label-free methods (Raman spectroscopy (SERS) [198, 199], atomic force microscopy, etc.) as well as label-based methods (nano-flow cell technology [200, 201], microfluidic chip [202], etc.). Although current technologies are limited to the detection of known proteins, they hold broad application prospects as technology advances and detection panels expand.

The glycosylation of EV surface proteins not only affects their stability, targeting, and cellular uptake capabilities but also demonstrates great potential in disease-specific recognition and biomarker discovery [203]. The major forms of glycosylation include N-linked and O-linked glycans. By structurally characterizing the glycan chains on EV surfaces, disease-associated specific glycoforms can be identified, aiding in precise diagnosis. Recent studies have shown that EV glycosylation plays a key role in their biogenesis, membrane protein loading, and secretion processes, and is closely associated with various disease states [204].

Glycobiological approaches have also been used. By using various types of lectins as probes, specific glycoform-enriched EV subpopulations can be identified and isolated, offering new strategies for liquid biopsy in multiple cancers [205–208]. Furthermore, glycoproteomics combined with mass spectrometry has become one of the mainstream tools for studying EV glycosylation, enabling precise mapping of disease-associated glycosylation profiles [209, 210].

### Machine learning applications based on circulating EV metabolomics features

Studies have shown that novel material platforms (such as composite nanoprobes and core-shell nanoparticles) can efficiently capture EVs and directly obtain their metabolic fingerprints, combined with ML algorithms, to achieve accurate identification of endometrial cancer, hepatocellular carcinoma, liver cirrhosis and other diseases [211, 212]. Through mass spectrometry, these studies further identified disease-related characteristic metabolites, highlighting the potential application of vesicular metabolomics in liquid biopsy.

## Machine learning applications based on multi-omics joint features

The complexity of tumor biology is such that single-omics analyses can only reveal local features of the mechanism. At the same time, the integration of multi-omics data (cross-molecule type integration) and multimodal features (incorporating clinical characteristics) can provide a more comprehensive view of disease diagnosis, treatment monitoring, and prognosis assessment. The integration of multi-source data and systematic analysis of multi-dimensional tumor characteristics is enabled by advanced computing power and ML algorithms, which, in turn, improves marker screening and model prediction [51].

Regardless of whether there is membrane structural encapsulation or not, joint analysis of paired data from different sources as potential biomarkers is a worthwhile strategy. Cross-molecule type integration combines biomarkers from different sources, such as combined analysis of free and vesicle-derived RNA or DNA, effectively improving the ability to identify diseases such as pancreatic cancer, even in people who are negative for traditional markers [213, 214].

Additionally, fusing novel molecular markers with clinically validated traditional biomarkers constructs prediction models with enhanced stability and broader population coverage, addressing the limited diagnostic performance of single marker types [215].

Furthermore, the incorporation of patient clinical features (e.g., age, gender, and primary tumor site) has been demonstrated to enhance the performance of diagnostic and prognostic models [216] and the discrimination ability of the model in scenarios such as benign and malignant tumors [91, 217].

## Challenges and responses in the clinical rollout of hematologic marker models

**Fig. 6:**
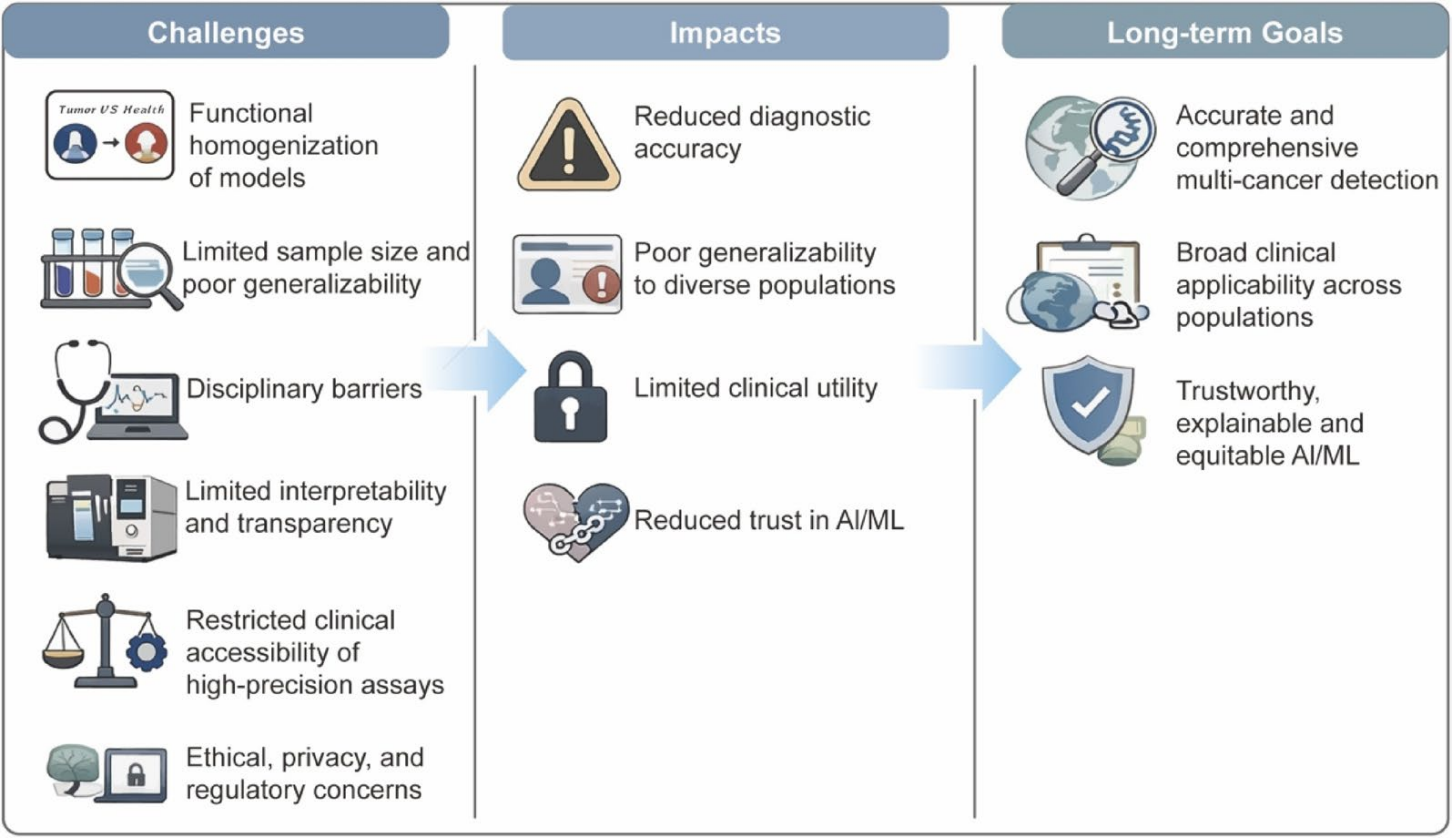
Overview of challenges, impacts, and long-term goals of AI/ML-driven liquid biopsy biomarkers for cancer diagnosis. Left: Key challenges, including model homogenization, limited sample size, disciplinary barriers, poor interpretability, restricted assay accessibility, and ethical/privacy concerns. Middle: Impacts of these challenges, including reduced diagnostic accuracy, poor generalizability, limited clinical utility, and weakened trust in AI/ML. Right: Long-term goals, including multi-cancer detection, broad clinical applicability, and the development of trustworthy, explainable, equitable AI/ML systems

Despite the evident potential of ML in the prediction of serum markers and the development of diagnostic models, the field continues to face significant challenges. The biomarkers identified in the majority of studies remain constrained to laboratory validation. This review focuses on clinical translation barriers and systematically identifies key bottlenecks beyond model performance and the paths to address them (Fig. 6).

1. Model functionality homogenization: The current ML models of cancer liquid biopsy mainly focus on binary classification (tumor patients vs. healthy people). It is not due to the inherent decline of multi-classification model performance, but the distortion of multi-label data of liquid biopsy (such as low data quality, high feature dimension, and high overlap of features of different cancer types), which makes it difficult to establish an effective label assignment axis and affects the performance of multi-classification models [33, 218–220]. The core of improving the performance of multi-classification models is not to limit the number of model classifications, but to optimize the quality of multi-label data (such as improving the detection accuracy and reducing feature overlap). The multi-label classification algorithm is combined with the clinical characteristics of cancer to achieve accurate classification of multiple cancer types [221].
2. Insufficient sample size: Existing studies are often limited by small sample size (tens to hundreds of cases) and single source (single center/region), resulting in insufficient model generalizability. Some studies have used non-targeted histology to analyze small discovery cohorts and then switched to targeted assay technologies (e.g., qRT-PCR, ELISA) during the validation phase, which reduces the cost but introduces feature bias. In addition, blood markers are susceptible to confounding by diet, age, and other factors [222], compounded by issues such as difficult sample collection and high testing costs that severely limit clinical application [223, 224]. Therefore, it is essential to establish standardized processes to reduce technical differences and establish a data sharing and research collaboration platform. Fehlmann’s team evaluated the diagnostic efficacy of lung cancer by collecting genome-wide miRNA profiles of 3,000 cases from more than 10 centers [223]; Circulating Cell-free Genome Atlas (CCGA) project integrated 15,000 samples from more than 140 regions to establish a cfDNA database [90]; and the China-led International Collaboration Program on Human Proteome Navigation (π-HuB) is dedicated to global proteome data sharing to promote the development of precision medicine [225].
3. Disciplinary barriers: Disciplinary silos between clinicians and data scientists remain a key barrier to translation, which can be addressed through interdisciplinary collaborative teams and user-friendly code-free analytical platforms.
4. Model interpretability and transparency: Machine-learning models suffer from a tradeoff between interpretability and performance: simple models are easy to interpret but limited in their ability to handle complex data, whereas high-performing complex models have low clinical confidence because of their “black-box” nature. The EU General Data Protection Regulation (GDPR) requires transparent explanations of the logic and implications of automated decisions. Model interpretability not only enhances doctor-patient trust and optimizes marker screening, but also helps to identify potential biomarkers of high diagnostic value [52, 226–229]. There are two main types of interpretable methods: endogenous interpretable models are transparent but limited; Post-processing techniques approximate the decision mechanism of a complex model through external tools, but their interpretation may deviate from the internal model logic. For high-dimensional cfDNA fragmentomics data, although SHAP can provide a consistent ranking of feature importance, its computational complexity increases sharply with the increase of feature dimension, and there is a significant computational efficiency bottleneck when processing genome-wide fragmentomics features. This limits its application in large cohorts or clinical scenarios where rapid feedback is required [102, 103]. LIME, on the other hand, provides explanations by building surrogate models locally, but the stability of its explanations depends heavily on the generated perturbation samples. In EV-RNA or EV-proteomics studies with small samples, the limited sample size makes the generated perturbation data difficult to truly reflect the original data distribution, which leads to large deviations and even contradictions in the interpretation results, reducing the reliability of its interpretation [194, 195]. In the future, a balance between performance and interpretability must be sought to increase clinical confidence and the potential for widespread application [228, 230–232].
5. Limitations of high-precision assays: Mass spectrometry and next-generation sequencing (NGS) limit clinical applications due to high costs [145]; while EV marker analysis, such as ultracentrifugation and size-exclusion chromatography, limits feasibility in large-scale clinical applications due to operational complexity and lack of reproducibility [233–235]. Therefore, there is an urgent need to develop more cost-effective and reproducible assay techniques [236–240]. Generally, novel and convenient methods are based on immunocapture for direct capture in serum or plasma, and Huang et al. developed the CRC-EVArray microarray for direct capture of sEVs and specific surface proteins in trace amounts of serum, which overcomes the limitations of traditional techniques that require large amounts of body fluids. Zhang et al. used functionalized magnetic beads to rapidly and efficiently separate sEVs and improve protein identification [50]. Cao et al. validated the advantages of chitosan-magnetic bead-based separation (Mag-CS) for EV separation by comparing and evaluating the effectiveness of different methods for sEV separation in serum [241]. In addition, there are a large number of portable testing devices whose research and development is driving the technology to iterate towards low cost and high efficiency, providing a new pathway for clinical scale-up [242–244].
6. Ethical, privacy and regulatory challenges: Hematology marker research relies on a large amount of data from patient and healthy human samples and faces serious ethical and privacy challenges, and urgently needs dynamic updates of regulations and guidelines to ensure safe data use. In the future, similar technologies such as data de-identification techniques will play an important role in model development and application [245].

## Future perspectives

Non-invasive diagnostic technologies based on liquid biopsy have rapidly advanced due to increasing clinical demand. In recent years, rapid progress in technologies capable of ‘reading’ biological information has enabled researchers to readily acquire extensive biological and genetic data. Simultaneously, the swift development of AI has offered researchers the opportunity to identify effective biomarkers from large, complex, and unstructured datasets, thereby facilitating the construction of diagnostic and prognostic models. This review summarizes the evolution of ML and its contributions to biomarker discovery in liquid biopsy.

Future diagnostic improvements will hinge on two fronts: fundamental research into liquid biopsy analytes—such as novel EV subsets and migrasomes—coupled with innovative sequencing strategies, and enhanced AI capabilities for interpreting multi-omics and multi-dimensional data to build robust diagnostic models.

Looking forward, clinical breakthroughs in liquid biopsy technology require multidisciplinary collaboration, including fundamental breakthroughs in the biology of liquid analytes, multi-omics data integration, optimization of ML algorithms, and advancements in detection technologies such as SERS. Collectively, these efforts promise to accelerate early disease screening and advance precision medicine through prospective validation.

## Data Availability

No datasets were generated or analysed during the current study.
